# On-line solid-phase enrichment coupled to packed reactor flow injection analysis in a green analytical procedure to determine low levels of folic acid using fluorescence detection

**DOI:** 10.1186/1752-153X-6-155

**Published:** 2012-12-12

**Authors:** Samy Emara, Tsutomu Masujima, Walaa Zarad, Maha Kamal, Ramzia EL-Bagary

**Affiliations:** 1 Pharmaceutical Chemistry Department, Faculty of Pharmacy, Misr International University, Km 28 Ismailia Road, Cairo, Egypt; 2 Analytical Molecular Medicine & Devices Laboratory, Hiroshima Univ., Graduate School of Biomedical Sciences, 1-2-3, Kasumi, Minami-ku, Hiroshima, 734-8551, Japan; 3 Pharmaceutical Analytical Chemistry Department, Faculty of Pharmacy, Modern Sciences and Arts University, 26 July Mehwar Road intersection with Wahat Road, 6 October City, Egypt; 4 Pharmaceutical Chemistry Department, Faculty of Pharmacy, Cairo University, Kasr El Aini St, Cairo, 11562, Egypt

**Keywords:** Folic acid, On-line solid phase enrichment, Flow injection analysis, Cerium (IV) trihydroxyhydroperoxide, Fluorescence detection

## Abstract

**Background:**

Analysis of folic acid (FA) is not an easy task because of its presence in lower concentrations, its lower stability under acidic conditions, and its sensitiveness against light and high temperature. The present study is concerned with the development and validation of an automated environmentally friendly pre-column derivatization combined by solid-phase enrichment (SPEn) to determine low levels of FA.

**Results:**

Cerium (IV) trihydroxyhydroperoxide (CTH) as a packed oxidant reactor has been used for oxidative cleavage of FA into highly fluorescent product, 2-amino-4-hydroxypteridine-6-carboxylic acid. FA was injected into a carrier stream of 0.04 M phosphate buffer, pH 3.4 at a flow-rate of 0.25 mL/min. The sample zone containing the analyte was passed through the CTH reactor thermostated at 40°C, and the fluorescent product was trapped and enriched on a head of small ODS column (10 mm x 4.6 mm i.d., 5 μm particle size). The enriched product was then back-flush eluted by column-switching from the small ODS column to the detector with a greener mobile phase consisting of ethanol and phosphate buffer (0.04M, pH 3.4) in the ratio of 5:95 (v/v). The eluent was monitored fluorimetrically at emission and excitation wavelengths of 463 and 367 nm, respectively. The calibration graph was linear over concentrations of FA in the range of 1.25-50 ng/mL, with a detection limit of 0.49 ng/mL.

**Conclusion:**

A new simple and sensitive green analytical procedure including on-line pre-column derivatization combined by SPEn has been developed for the routine quality control and dosage form assay of FA at very low concentration level. The method was a powerful analytical technique that had excellent sensitivity, sufficient accuracy and required relatively simple and inexpensive instrumentation.

## Background

**Figure 1:**
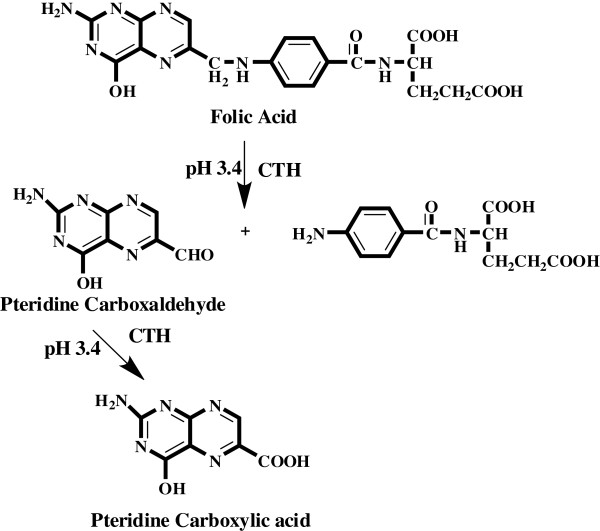
Structures of the investigated compounds.

Folic acid (FA) (N(4-((2-amino-l,4-di-hydro-4-oxo-6-pteridinylmethyl)amino)-benzoyl)-L-glutamic acid) (Figure 1), which occurs naturally in cereals, is essential in humans. FA plays a major role in the synthesis of red blood cells, in the formation of RNA and DNA, in the development of tissues and the brain of the fetus and the growth of a baby [1].

Several analytical methods have been developed for the determination of FA, including spectrophotometry [2,3], fluorimetry [4-7], chemiluminescence [8], enzyme assay [9], capillary electrophoresis [10,11] and high-performance liquid chromatography (HPLC) [12-14]. The United State Pharmacopoeia for the determination of FA is based on HPLC with UV detection at 254 nm [15].

In recent years, more strict regulation related to the quality control of pharmaceuticals led to increasing demands on automation of the analytical assays carried out in appropriate control laboratories. The flow injection analysis (FIA) technique became a versatile instrumental tool that contributed substantially to the development of automation in pharmaceutical analysis due to its simplicity, low cost and relatively short analysis time. A survey of the literatures reveled that there are few FIA methods for the determination of FA in pharmaceutical formulations. Chemiluminescence (CL) FIA assay for FA has been reported [16]. The CL system was based on inhibition of the chemiluminescent reaction of ferricyanide and luminol by FA in alkaline medium. Ferricyanide and luminol have also been immobilized on an anion-exchange column in FIA and used for CL inhibition assay of FA [17]. Wang et al., [18] performed an analysis of FA by FIA with fluorescence detection using ferricyanide solution in alkaline sodium hydroxide and methanol. Lead dioxide solid-phase reactor incorporated into FIA manifold for oxidation of FA into a fluorescent derivative has been employed to determine the analyte in pharmaceuticals [19]. A CL method for the determination of FA by the sodium hypochlorite and semicarbazide hydrochloride system with a FIA technique has been reported [20]. Recently, Zhao and co-workers have reported a CL-emitting reaction between potassium tetrabromoaurate (III) and luminol in alkaline medium. This reaction has been incorporated with FIA and off-line solid-phase extraction for the determination of FA [21].

Determination of low levels of drugs is an important issue in quality control of pharmaceuticals. Appropriate selection of pre-concentration method for on-line sample processing and suitable detection allows the development of FIA methods for such analysis. In addition, the potential to develop green analytical procedures is inherent to flow analysis, and changes in system design as well as the exploitation of new flow approaches have led to ingenious alternatives to minimize reagent consumption and waste generation without hindering analytical performance. Therefore, our study was involved in a research effort aiming to develop and validate a green analytical procedure including on-line derivatization combined by column switching with ODS small column for solid-phase enrichment (SPEn) to determine FA. The method was based on oxidative cleavage of FA into highly fluorescent 2-amino-4-hydroxypteridine-6-carboxylic acid (Figure 1) using a packed reactor of cerium (IV) trihydroxyhydroperoxide (CTH) and the reaction product was enriched on small column. The trapped fluorescent derivative was back-flush eluted to fluorescence detector with an environmentally friendly mobile phase consisting of phosphate buffer and ethanol. High reagent consumption is the main disadvantage of all continuous FIA systems [16-21], which use the reagent continuously even when no sample is present in the apparatus. In the present work, the packed reactor avoids this uneconomical approach in such a way that the sample zone meets the CTH reactor in a controlled manner while the rest of the system is filled with 0.04 M phosphate buffer, pH 3.4. Also, simplifications of manifold and improved mixing conditions are provided by the CTH packed reactor. According to previous studies with methotrexate, CTH solid reactor has been selected for derivatization of FA because this reagent showed good reproducible results [22]. However, the implementation of on-line SPEn with FIA-manifold in the present work provides enhanced sensitivity over 20 times, that makes FIA-SPEn strategy promising for determination of FA in pharmaceutical formulation down to a level of 0.49 ng/mL.

### Experimental

#### Instrumentation

**Figure 2:**
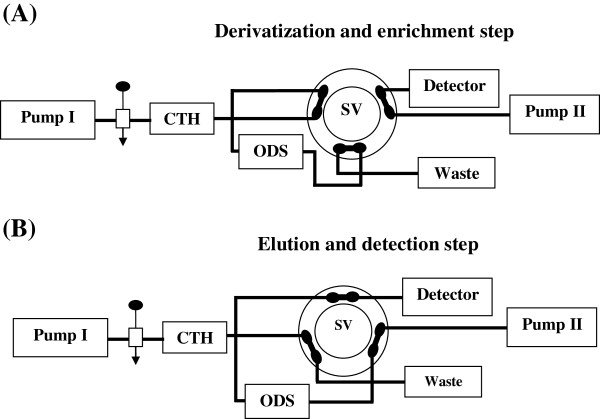
Schematic diagram of on-line-SPEn coupled to packed reactor FIA for the analysis of FA in pharmaceutical formulations: the system in initial position, ready for sample injection, derivatization and enrichment step (A); the back-flush elution and detection step (B) (S.V.: switching valve).

A single line FIA-SPEn manifold, illustrated in Figure 2, consisted of two solvent delivery pumps (Agilent 1100 Series Iso pump G1310A). One was used to deliver the carrier solution at a flow-rate of 0.25 mL/min and the other delivered the isocratic mobile phase at a flow-rate of 1 mL/min. This system was equipped with two columns; one was a short (70 × 4.6 mm i.d.) CTH packed oxidant column for oxidative-cleavage of FA into highly fluorescent product and the other was a small TSK gel ODS-80 TM column (10 × 4.6 mm i.d., 5 μm particle size) for pre-concentration of the fluorescent product. A model 7125 sample injection valve (400 μL) and a model 7010 flow direction switching valve were applied to load the sample onto the CTH packed oxidant, to facilitate oxidative cleavage of FA into highly fluorescence product and to elute the enriched analyte in a back-flush elution mode to the detector, respectively (Rheodyne, Berkeley, CA, USA). The eluent was monitored by a fluorescence detector, (Agilent 1200 series, G1321A) set at an excitation wavelength of 367 nm and an emission wavelength of 463 nm. Data acquisition was performed on Agilent LC ChemStation software. The ODS column temperature was ambient, and that of the CTH-packed reactor was 40°C.

### Reagents and chemicals

FA (99.81% purity) was obtained from Kyowa Hakki (Tokyo, Japan). The present FIA method was applied to the determination of FA in its pharmaceutical formulations: FA tablet (Batch No. 940111) was manufactured by Mepaco Medifood Enshas, Sharkeya, Egypt. Each tablet was claimed to contain 5,000 μg of FA. The ethanol was HPLC grade (BDH, Poole, UK). Distilled water was used for the preparation of all reagents and solutions. Potassium dihydrogen phosphate, ortho-phosphoric, sodium hydroxide, hydrochloric acid, chloroform and isopropyl alcohol were analytical grades. TSK gel ODS-80 TM (5 μm) was from Tosoh Corporation (Tokyo, Japan).

### Mobile phases

Two different mobile phases were employed in the assay procedure. One was 0.04 M phosphate buffer, pH 3.4 which was used as a carrier stream (**MI**) to deliver the sample to the CTH packed oxidant in the oxidation step. The other was an isocratic solvent system (**MII**) consisting of ethanol and phosphate buffer (0.04 M, pH 3.4) (5:95 v/v) and used to elute the enriched fluorescent product in a back-flushed mode from the head of the small ODS column to the fluorescence detector. All mobile phases were freshly prepared on the day of use, filtered through 0.45 μm filters and degassed ultrasonically under vacuum.

### Preparation of ODS and CTH columns

The ODS (5 μm TSK gel ODS-80 TM) and CTH packing materials were suspended in chloroform and isopropyl alcohol, respectively. The suspended materials were degassed under vacuum with continuous stirring for 10 min. A stainless-steel cylinder (100 × 4.6 mm i.d.) was used as a reservoir for the packing materials. This reservoir was connected to a short column (10 × 4.6 mm i.d.) for ODS materials and another column (70 × 4.6 mm i.d.) for CTH packed oxidant. The suspended ODS and CTH supplied from the reservoir were packed into the corresponding columns with the aid of an HPLC pump at flow-rate of 5 mL/min with ethanol as a purge solvent (10 min). Pumping must continue until a constant pressure is reached. The cylinder was then disconnected and a mixture of ethanol and distilled-deionized water (1:1) was passed through the columns at a flow-rate of 1 mL/min for a further 10 min. The columns were then equilibrated with of 0.04 M phosphate buffer (0.04 M, pH 3.4) at a flow-rate of 1 mL/min for 30 min.

### General procedure

A 400 μL aliquot of FA sample was loaded into the injection valve and then injected into **MI**. The moving zone of FA passed through the CTH packed reactor at a flow-rate of 0.25 mL/min (pump I). The oxidative cleavage of FA occurs during the flow of **MI** containing the drug through the CTH column. Pre-concentration was performed by means of the flow of the fluorescent product from the packed oxidant column to the short ODS column head. After 4 min the valve was switched into position B (Figure 2). At this position, the **MII** could pass through the ODS column, where the fluorescent product was eluted in a back-flush mode to the detector. The flow-rate was maintained at 1 mL/min and the fluorescence intensity of the eluting compound was monitored at emission and excitation wavelengths of 463 and 367 nm, respectively. At 5 min after injection, the valve was switched into position A.

### Standard solution and calibration

FA (2.5 mg) was transferred to a 200 mL conical flask together with 150 mL distilled water. To promote the solution of FA, two drops of 1 M NaOH were added. Immediately after solvation, pH was adjusted to 7–8 with 0.1 M hydrochloric acid and quantitatively transferred to a 250 mL calibrated flask and made up to 250 mL with distilled water to produce stock standard solution of 10 μg/mL. A known volume of the stock standard solution was diluted with the same solvent to obtain a concentration of 1 μg/mL FA (solution A). The standard solutions for calibration were prepared daily by serial dilutions of appropriate volumes of solution A to produce FA standard solutions in the concentration range of 1.25-50 ng/mL. An aliquot of 400 μL was analyzed for FA according to the proposed procedure. The stock standard solutions of FA were stored in a dark flask at −20°C and were found to be stable for at least one month. The standard solutions for calibration were freshly prepared and stored in a dark flask at 5°C during use.

### Determination of FA in tablets

A total of 20 tablets containing FA as the active ingredient were weighed and powdered. A portion of the powder equivalent to 2.5 mg of FA was accurately weighed and transferred to a 200 mL conical flask with 150 mL distilled water. Two drops of 1 M NaOH were added, and the resulting solution was sonicated for 30 min. pH was adjusted to 7–8 with 0.1 M hydrochloric acid and the solution was quantitatively transferred to a 250 ml calibrated flask and completed to a 250 mL with distilled water to produce stock standard solution of 10 μg/mL; it was then filtered through a 0.45 μm membrane filter. The first portion of the filtrate was discarded and the remainder was used as a stock sample solution (Solution A, 10 μg/mL). A known volume of solution A was diluted quantitatively with distilled water to obtain a concentration of 20 ng/mL FA. An aliquot of 400 μL was analyzed for FA according to the proposed procedure. The sample solution was stored in a dark flask at 5°C during use.

## Result and discussion

The use of a packed reactor, in the described FIA-SPEn assembly, as an alternative to existing reagent solutions for the determination of FA was dependent on optimization of the system to achieve the maximum detector response. On the basis of experimental results, it could be stated that the pH, concentration and flow rate of carrier stream, sample volume and packed reactor temperature were the key parameter to improve derivatization step (position A). The other parameters optimized were: pH and buffer concentration as well as concentration of ethanol used as organic modifier in the elution step (position B).

### Effect of pH and concentration of carrier stream

**Figure 3:**
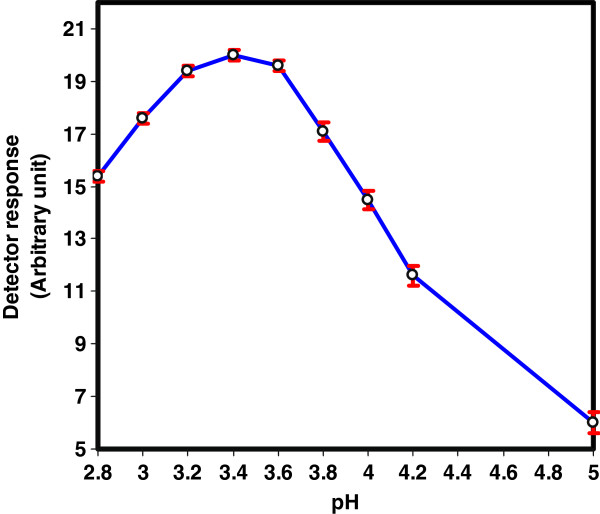
Effect of pH of carrier stream on the reaction efficiency of CTH packed reactor using 0.04 M phosphate buffer at a flow rate of 0.25 mL/min and packed reactor of temperature 40°C.

Carrier stream pH is a very important chemical parameter that has to be investigated. The correct adjustment of this variable is necessary to improve the reaction completeness between the analyte and CTH packed reactor. The influence of this parameter on the analytical signal was studied in the pH range of 2.8-5 using 0.04 M phosphate buffer at a flow rate of 0.25 mL/min and packed reactor temperature of 40°C. The analytical signal reaches its maximum value in the narrow range of pH 3.2-3.6 (Figure 3). At pH < 3.0, a low signal response was observed which might suggest the decomposition of the peroxy groups of the CTH materials, whereas at pH > 3.8, the detector signals was decreased abruptly probably due to the low CTH reactor efficiency which reduces drastically the oxidative cleavage of FA into highly fluorescent derivative. Accordingly, phosphate buffer solution of pH 3.4 was selected as the optimum carrier stream.

**Figure 4:**
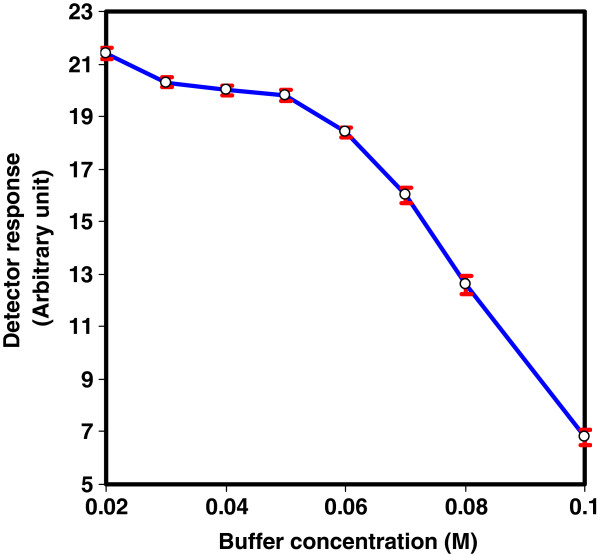
Effect of phosphate buffer concentration on the reaction efficiency of CTH- packed reactor using pH 3.4 a at flow rate of 0.25 mL/min and packed reactor temperature of 40°C.

To find out the suitable salt concentration, phosphate buffer (pH 3.4) of concentrations varying from 0.02 to 0.1 M were examined at a flow rate of 0.25 mL/min and packed reactor temperature of 40°C. Best analytical signals were verified within the concentration range 0.02-0.05 M (Figure 4). Above this range, the peak area that corresponds to the analyte was significantly decreased probably due to greater quenching effect on the fluorescence signal intensity. Although, 0.02 M phosphate buffer showed slight improvement of signal intensity, 0.04 M was chosen as a compromise between detector response and precision.

### Effect of the flow rate

The reaction of CTH packed reactor with FA was highly influenced by the flow-rate of carrier stream. To achieve a substantial enhancement of the detector response, the effect of flow-rate on the fluorescent intensity of FA was investigated in the range of 0.25-2.5 mL/min using phosphate buffer (0.04 M, pH 3.4) as a carrier stream and packed reactor temperature of 40°C. Higher flow-rate could cause shorter residence time (reaction time) and incomplete reaction for the target analyte with CTH. On the other hand, lower flow-rate could cause higher dispersion of sample zone over a wider area of the solid CTH and the reaction was proceeded sufficiently. Unlike previously reported assay using the conventional packed reactor FIA approach [22], a lower flow-rate was justified in the present work for the determination of FA because peak enrichment could be achieved on the top of the small ODS column. As far as the oxidation reaction proceeded, 2-amino-4-hydroxypteridine-6-carboxylic acid could be accumulated on the top of the ODS column with a zone width almost independent on the flow-rate. A compromise between analytical signal and sample frequency was established by choosing a working flow-rate of 0.25 mL/min. However, it should be taken into account that flow-rate less than 0.25 mL/min reduces the sampling frequency.

### Effect of temperature

**Figure 5:**
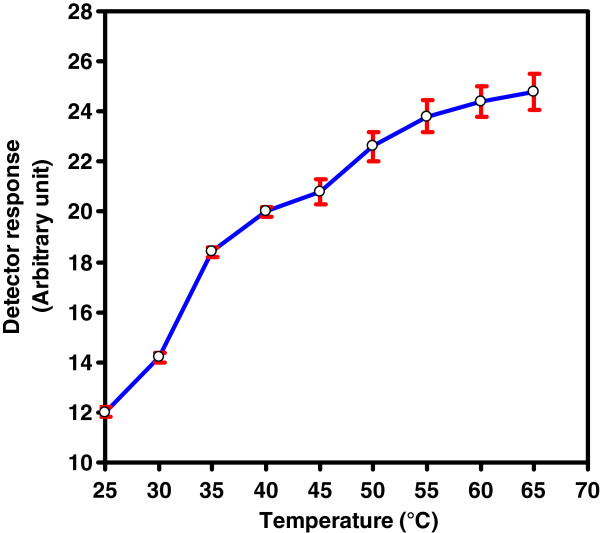
Effect of temperature on the reaction efficiency of CTH-packed reactor using phosphate buffer (0.04 M, pH 3.4) as a carrier stream at a flow rate of 0.25 mL/min.

The effect of temperature, for the oxidative cleavage of FA with CTH packed reactor, was examined by thermostating the packed reactor in the range from 25 to 65°C using phosphate buffer (0.04 M, pH 3.4) as a carrier stream at a flow-rate of 0.25 mL/min. The results showed that the reaction of FA with CTH could be proceeded at room temperature (25°C) while the detector response quickly increased along with an increase in the temperature, which might mean that high temperature accelerates the oxidation reaction of FA with CTH (Figure 5). It was observed that heating above 45°C resulted in an increase of column pressure and showed a generally drastic effect on the CTH packed oxidant life span. Considering the effective reaction temperature and the packed oxidant limitations, 40°C was selected as the optimum value because under this condition good sensitivity and reproducibility were achieved.

As a result, the optimal oxidation reaction efficiency of the CTH packed reactor could be achieved by using 0.04 M phosphate buffer, pH 3.4 at a flow-rate of 0.25 mL/min and 40°C.

### Effect of sample volume

One of the advantages of on-line SPEn is the potential increase in the sensitivity by increasing the sample volume used for analysis of FA due to the pre-concentration of a higher amount of fluorescent product on a head of small ODS column before detection. Therefore, choosing an appropriate sample size has a large effect on the ability to detect and quantify FA at lower levels. Different injection volumes (50–500 μL) were tested to introduce decreasing concentration of FA. The efficiency of enrichment for fluorescent product was evaluated on the basis of the linearity of calibration curve constructed over sample volumes (50-400μL, at 50 μL interval). It was found that, the CTH packed oxidant could tolerate large volumes of FA standard solution and the linear relationship between the peak area and the injected volumes was observed over the range of 50–400 μL sample volume. The regression equation for the influence of the sample volume on the analytical signal in this range was *Y* = 0.1878*V* + 0.4610 (*r* = 0.9992), where *Y* is the peak area and *V* denotes the loaded volume in μL of FA. If too large a sample volume is used (more than 400 μL), then the linearity between the peak area and concentration of FA will be disturbed because a long residence time was found necessary to get reproducible results upon using larger volumes. Accordingly, sample volume of 400 μL FA sample was selected as a compromise between the sensitivity and accuracy.

### Breakthrough study of ODS small column

Insertion of a small ODS column in the FIA manifold is very useful for enrichment of the analyte before detection. For optimal on-line pre-concentration conditions, with respect to band broadening, the retention of the fluorescent product should be high at enrichment mode and low at elution mode. In the present investigation, TSK gel ODS-80 TM silica was chosen to be suitable packing materials for the preparation of easily replaceable short column (10 × 4.6 mm i.d., 5 μm particle size). A breakthrough study was performed by injecting 400 μL sample containing FA (20 ng/mL) onto the CTH column, by means of **MI**. Increasing volume of **MI** was pumped through the column, before the switching valve was switched to position (**B**) and the trapped product was brought to the fluorescence detector with the analytical mobile phase (**MII**). The results indicated that, with up to 10 mL of phosphate buffer (0.04 M, pH 3.4), the recovery of the drug was above 99%, which clearly showed that there was no risk of losing FA during the enrichment step.

### Optimization of the back-flush elution step

In the elution step (position B) (Figure 2), the trapped fluorescent product was back-flush eluted by column-switching from the ODS column using **MII.** Thus, the time needed to elute the fluorescent product from the ODS column and to transfer it to the detector was tested. For this reason, we have studied the influence of pH, buffer and ethanol concentrations on the retention behavior of the fluorescent product. A green mobile phase consisting of ethanol and phosphate buffer (0.04M, pH 3.4) in the ratio of 5:95 (v/v) was chosen as a good elution mobile phase (**MII**). Due to the elution strength of **MII**, a time period of less than 1 min was sufficient to perform the complete transfer of fluorescent product from the head of a short ODS column (10 × 4.6 mm i.d.) to the detector. By this means, much faster analysis was possible and the productivity of the proposed FIA-SPEn method could be increased.

### Method validation

#### Linearity

**Figure 6:**
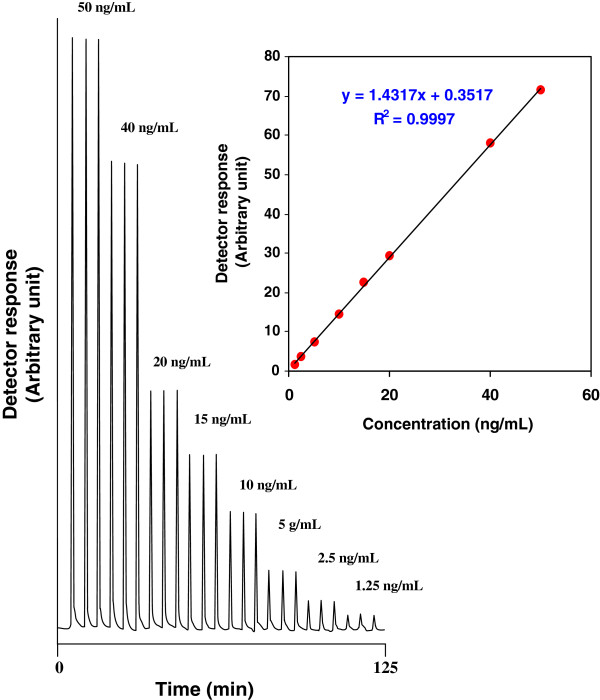
**Diagram chart and calibration curve (inset) for on-line pre-column derivatization combined by SPEn to determine FA over the concentration range of 1.25-50 ng/mL.** Experimental conditions: phosphate buffer (0.04M, pH 3.4) at a flow-rate of 0.25 mL/min and CTH reactor temperature of 40°C in derivatization and enrichment step; mobile phase consisting of ethanol and phosphate buffer (0.04M, pH 3.4) in the ratio of 5:95 (v/v) at a flow-rate of 1 mL/min for back-flush elution step.

Calibration curve was constructed by plotting the measured peak area of FA versus concentration over the concentration range of 1.25-50 ng/mL. Each concentration was repeated three times; this approach provided information on the variation in peak area values between samples of same concentration. The linearity of the calibration curve was validated by the high value of the correlation coefficient (0.9997). The equation for the best-fit straight line was determined by the linear regression analysis as Y = a + bC, where *Y* is the peak area and *C* denotes the concentration in ng/mL of FA. Characteristic parameters of the linear calibration curve are shown in Table 1. Diagram chart of experimental peaks at different concentration of standard solutions and calibration curve (inset) for on-line pre-column derivatization combined by SPEn to determine FA is shown in Figure 6. The limit of detection (LOD) was determined according to ICH guidelines for validation of analytical procedures [23] and was found to be 0.49 ng/mL (Table 1). 

**Table 1:** Linearity parameters and intra- and inter-day validation of the method to determine FA in freshly prepared tablets

**Linearity parameters**^**a**^		**Concentration (ng/mL)**	**Intra-assay**	**Intra-assay**
**Calibration range (ng/mL)**	**1.25-50**		**Mean recovery**^**b**^**(% ± RSD)**	**Mean recovery**^**b**^**(% ± RSD)**
Detection limit (ng/mL)	0.49	5	98.88 ± 0.62	98.45 ± 0.81
Slope (b)	1.4317	15	99.15 ± 0.34	99.01 ± 0.44
Standard error of the slope	0.0094	30	99.27 ± 0.44	99.13 ± 0.53
Intercept (a)	0.3517	45	99.14 ± 0.51	98.92 ± 0.59
Standard error of the intercept	0.2319		Average	
Correlation coefficient (*r*^2^)	0.9997		99.11 ± 0.48	98.88 ± 0.59

^a^ Y = a + bC, where C is the concentration of FA in ng/mL and Y is the peak area.

^b^ Values are mean of five determinations.

### Precision and accuracy

The precision of the analytical procedure was determined for both intra- and inter-day variations and expressed as the relative standard deviation (RSD%) of the mean measured concentration. Repeatability (intra-day RSD, *n* = 5) and reproducibility (inter-day RSD, *n* = 5) was excellent being in the ranges of 0.34-0.62% and 0.44-0.81%, respectively. Repeatability and reproducibility of FA samples with high and low concentration levels were below 1.00%, indicating a reliable measurement using the proposed method (Table 1). The accuracy of the method was assessed by determining analytical recovery. Recoveries for all real samples were in the range of 99.27–98.88% for intra-day and 99.13–98.45% for inter-day studies. The average recovery values were 99.11 and 98.88% with RSD values of 0.48 and 0.59% for intra- and inter-assay precision, respectively (Table 1). These good recovery values and low RSD values revealed the high accuracy and precisions, of on-line derivatization-SPEn strategy, respectively.

### Column lifetime

The column lifetime, in terms of its ability to quantitatively derivatize FA to give highly fluorescent product, was investigated as a function of the volume of samples (20 ng/mL, FA) injected onto the CTH-packed reactor using phosphate buffer (0.04M, pH 3.4) as a carrier stream at a flow-rate of 0.25 mL/min and packed reactor temperature of 40°C. It was found that this solid reactor could successfully be used for loading 100 mL samples. Increasing the injection volumes above this level led to a decrease in sensitivity and an increase of the column back pressure with final clogging. Accordingly, a CTH column should be renewed when it shows excessive backpressure or lower efficiency. Repetitive injections of 400 μL of FA (20 ng/mL), five times, each under continuous operation for 8 h every day over a period of 14 days, was conducted. It was found that the CTH column retained 99.15% of its efficiency over this period, after which a gradual pressure build-up at the head of the column was observed. The day to-day relative standard deviation for FA was found to be less than 4%.

### Interferences

To examine the selectivity of the proposed FIA-SPEn method, the effect of common excipients normally used in pharmaceutical formulations was studied. Solutions containing FA (20 ng/mL) in the presence of more than 100 folds of common additives such as magnesium stearate, sodium benzoate, xanthan gum, iron (II) sulphate, corn starch, lactose and sucrose were prepared. The undissolved materials were filtered off before injection. No significant changes were observed on the results and recoveries in the range of 99.18 - 99.32% were obtained in all cases.

### Practical applications of the proposed method

FA in commercial pharmaceutical formulations was determined by the proposed FIA-SPEn method and official USP method [15]. The results obtained by the FIA-SPEn procedure were in good agreement with those obtained by the official method (Table 2). The accuracy and precision of the developed method were further judged by applying *t*- and *F*-test at 95% confidence level. The experimental *t*- and *F*- values did not exceed the theoretical values, which support the similar accuracy and precision of the proposed and official methods (Table 2). The accuracy of the analytical method was also checked by standard addition method, which applied by adding drug standard to previously analyzed tablets. The accuracy shows that the derivatization procedure developed for the determination of FA can be considered as accurate within the concentration range investigated (Table 2). Mean value is very close to the theoretical concentration, showing method % recovery of 99.24 and RSD of ± 0.34. These results indicate that the effects of the common additives and ingredients of the pharmaceutical formulations do not interfere with the determination of FA.

**Table 2:** Determination of FA in commercial formulation by the proposed and official methods

**Concentration (20 ng/mL)**	**Recovery % **^**a**^**( ± RSD)**	
	**Proposed method**	**Official method**
Tablets	99.31 ± 0.30	99.56 ± 0.23
*t-*	0.29 (2.30) ^b^	
*F-*	1.74 (6.38) ^b^	
Recovery^c^ (%, ± RSD)	99.24 ± 0.34	

^a^ Mean Recovery % and RSD for five determinations.

^b^ Theoretical values for *t-* and *F-.*

^c^ For standard addition of 50% of the nominal content.

The proposed on-line derivatization-SPEn strategy is superior to other published FIA methods with respect to simplicity and sensitivity. The linearity range and detection limit were the parameters used to compare the sensitivity of the proposed method with the other reported FIA methods. The lowest reported linearity ranges (ng/ mL) of FA were found to be 10–15000 [17], 100–21000 [16], 8–2500 [19], 100–50000 [20] and 250–8000 [18], while the lowest detection limits (ng/mL) were 3.5 [17], 30 [16], 0.1 [19], 27 [20] and 130 [18]. In the developed method, the calibration plot showed excellent linearity with correlation coefficients of 0.9997 over the range from 1.25 to 50 ng/mL. The corresponding detection limit of FA was found to be 0.49 ng/mL. Although the detection limit of FA is higher than that reported by Zhao et al., (0.02 ng/mL) [21], the developed method has the advantages of simplicity and convenience. Intensive sample cleanup and enrichment by off line solid-phase extraction using cyclohexane followed by methanol and then distilled water was necessary to achieve the required selectivity and sensitivity in the reported method [21]. The sample cleanup procedure limited the ultimate performance of this method, especially with regard to ruggedness and reliability. Whereas, the implementation of on-line SPEn with FIA-manifold appears as a useful alternative to greatly decrease reagent consumption and the system is simplified with fewer junctions for mixing of reagents, sample and carrier streams. In addition, compared with the reported FIA method [19] for FA, the proposed procedure is worthy contributed to the existing environmentally friendly analytical chemistry due to reducing or elimination of the use and generation of hazardous substances.

### Robustness

To determine robustness of the proposed method, experimental conditions such as flow-rate, pH and concentration of the buffer solution used as a carrier stream, packed reactor temperature and organic content of the mobile phase were purposely altered and the detector responses were evaluated. Variation of each parameter by ±2% did not have a significant effect on the detector response.

## Conclusion

In this study, a green on-line derivatization-SPEn strategy using a packed oxidant reactor of CTH and a small ODS column has been developed for the first time to determine low levels of FA. The method was based on oxidative cleavage of FA into highly fluorescence product, 2-amino-4-hydroxypteridine-6-carboxylic acid followed by pre-concentration of the derivative on the head of the small column before detection. The high robustness of pre-column derivatization, good reproducibility, and simplicity made this method ideal for the routine quality control and dosage form assay of FA. Moreover, the proposed method was clearly superior in terms of sensitivity, which is particularly important in trace analysis when processing pharmaceutical samples. The applicability of the method was evaluated by the determination of FA in pharmaceutical formulations. Common excipients used as additives in pharmaceutical preparations did not interfere.

## Competing interests

The author(s) declare that they have no competing interests.

## Authors’ contributions

SE and TM suggested the idea of the assay and designed the proposed analytical method. SE supervised the whole study. WZ carried out the analytical experimental work, analyzed the data statistically, participated in the results and discussion, and preparing the manuscript. MK participated in the experiment work, assay design, results and discussion. REB participated in supervision and drafted the manuscript. All authors read and approved the final manuscript.
